# Distribution and antifungal susceptibility pattern of *Candida* species from mainland China: A systematic analysis

**DOI:** 10.1080/21505594.2022.2123325

**Published:** 2022-09-18

**Authors:** Hazrat Bilal, Muhammad Shafiq, Bing Hou, Rehmat Islam, Muhammad Nadeem Khan, Rahat Ullah Khan, Yuebin Zeng

**Affiliations:** a Department of Dermatology, The second Affiliated Hospital of Shantou University Medical College, Shantou, China; b Department of Cell Biology and Genetics, Shantou University Medical College, Shantou, China; c Department of laboratory, Shantou Municipal Skin Hospital, Shantou, China; d Key Laboratory of Space Bioscience and Biotechnology, School of Life Sciences, Northwestern Polytechnical University, Xi’an, China; e Faculty of Biological Sciences, Department of Microbiology, Quaid-i-Azam University, Islamabad, Pakistan; f Institute of Microbiology, Faculty of Veterinary and Animal Sciences Gomal University, Dera Ismail Khan, Pakistan

**Keywords:** *Candida*, antifungal susceptibility pattern, candidiasis, systematic review, china

## Abstract

Antifungal resistance to *Candida* pathogens increases morbidity and mortality of immunosuppressive patients, an emerging crisis worldwide. Understanding the *Candida* prevalence and antifungal susceptibility pattern is necessary to control and treat candidiasis. We aimed to systematically analyse the susceptibility profiles of *Candida* species published in the last ten years (December 2011 to December 2021) from mainland China. The studies were collected from PubMed, Google Scholar, and Science Direct search engines. Out of 89 included studies, a total of 44,716 *Candida* isolates were collected, mainly comprising *C. albicans* (49.36%), *C. tropicalis* (21.89%), *C. parapsilosis* (13.92%), and *C. glabrata* (11.37%). The lowest susceptibility was detected for azole group; fluconazole susceptibilities against *C. parapsilosis, C. albicans, C. glabrata, C. tropicalis, C. guilliermondii, C. pelliculosa*, and *C. auris* were 93.25%, 91.6%, 79.4%, 77.95%, 76%, 50%, and 0% respectively. Amphotericin B and anidulafungin were the most susceptible drugs for all *Candida* species. Resistance to azole was mainly linked with mutations in *ERG11, ERG3, ERG4, MRR1–2, MSH-2*, and *PDR-1* genes. Mutation in *FKS-1* and *FKS-2* in *C. auris* and *C. glabrata* causing resistance to echinocandins was stated in two studies. Gaps in the studies’ characteristics were detected, such as 79.77%, 47.19 %, 26.97%, 7.86%, and 4.49% studies did not mention the mortality rates, age, gender, breakpoint reference guidelines, and fungal identification method, respectively. The current study demonstrates the overall antifungal susceptibility pattern of *Candida* species, gaps in surveillance studies and risk-reduction strategies that could be supportive in candidiasis therapy and for the researchers in their future studies.

## Introduction

Antimicrobial resistance is a worldwide public health concern and is particularly worrisome regarding fungal infections. The fewer antifungals’ drugs for invasive infections and the emergence of multidrug-resistant (MDR) fungal pathogens have been associated with increased mortality. *Candida* species are the most common opportunistic pathogens that cause severe infections in the immunosuppressive host. The *Candida* species can cause superficial, vaginal, and oral mucosa infections and invasive candidiasis, such as deep tissue infections and bloodstream infections [1]. *Candida albicans* is the leading cause of candidiasis around the world. Besides this, the other common non-*C*. *albicans* species such as *C. parapsilosis*, *C. glabrata*, and *C. tropicalis* have emerged as health concerns over the past few decades [2]. In addition, the emergence and high prevalence of antifungal drugs resistant (AFR) *C. albicans* and MDR non-*C. albicans* species such as *C. auris*, have caused great concern for health care officials across the globe [1,3,4]. Phylogenetic analysis of *C. auris* shows five major clades that cluster geographically and are renowned for various single nucleotide polymorphisms [5,6]. Therefore, it is imperative to fully comprehend and monitor the trend of local epidemiology, and antifungal drug susceptibility to take smart decisions in the treatment of candidiasis [7].

The threat due to MDR *C. auris* further increases in the current COVID-19 epidemic as the ratio of hospitalized patients increases, providing a vulnerable environment for nosocomial infections. A recent study reported a mortality rate of 67.85% for coronavirus-associated *C. auris* infections [8]. Timely diagnosis and proper antifungal treatment are required to improve clinical outcomes and manage candidiasis [7,9]. However, laboratory-based technique like *Candida* culture is still inefficient for sensitive and rapid diagnosis of candidiasis. Therefore, the physicians most often prescribed empirical antifungal drugs [10,11]. The selection of empirical antifungal drugs mainly depends on epidemiological antifungal sensitivity data, which differs for every geographic region [3,7]. Hence, locally and country-wide antifungal surveillance data are required to select antifungal drugs accurately.

The molecular epidemiology of AFR *Candida* species is relatively less studied than the resistance mechanisms of bacteria [12]. The AFR *Candida* species needs to be addressed because of its high risk to human health as it ranked fourth amongst hospital-acquired and bloodstream pathogens [13]. In mainland China, the China Hospital Invasive Mycosis Surveillance Network (CHIF-NET) is an excellent project reporting antifungal susceptibility and epidemiology trends since 2009, functional in 30 out of 33 provinces (http://chifnet.com/login.cshtml). *C. albicans* is highly prevalent in China, but the proportion of emerging non-*C. albicans* species are increasing (reported >67% in 2020), mainly due to their AFR ability [14]. On this ground, several studies individually reported the epidemiology of AFR *Candida* species from various regions of China. However, there is no comprehensive systematic acquisition data on the antifungal susceptibility pattern of *Candida* from China. Therefore, in the current study, we planned to analyse the overall landscape of the distribution and antifungal susceptibility pattern of *Candida* from the published literature in China, which has not been documented. The outcomes of the present study will highlight the gaps in surveillance studies and provide recommendations for researchers [62]. Furthermore, the current study will provide directions for health care officials and prescribers about the resistance magnitude of *Candida* species to different antifungal agents, allowing them to devise strategies to combat and manage AFR in the region.

## Methods

### Literature search

**Figure 1.**
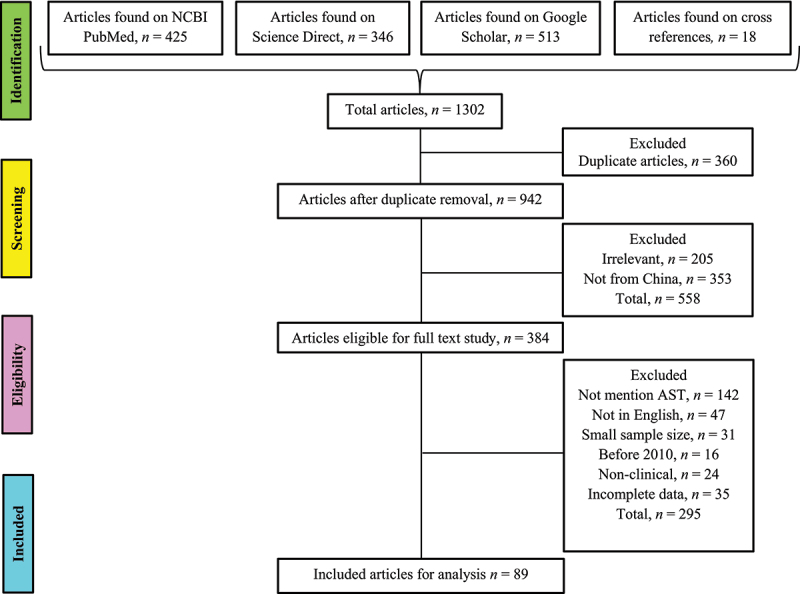
Studies identification and selection based on PRISMA guidelines.

The original research articles about *Candida* species in mainland China were collected from NCBI PubMed, Google Scholar, and Science Direct search engines. The preferred reporting items for systematic reviews and meta-analyses (PRISMA) guidelines and checklists were followed for article selection (Figure 1) [15]. The studies were searched by using keywords like; (“*Candida*” AND “Antifungal resistance” AND/OR “Antifungal susceptibility” AND China); (“Candidemia” AND “Antifungal resistance” AND/OR “Antifungal susceptibility” AND China) and (“Candidiasis” AND “Antifungal resistance” AND/OR “Antifungal susceptibility” AND China). Additionally, the bibliographies of selected research articles were thoroughly reviewed to access all the studies in the domain.

### Articles selections

All the articles found on the Databases were downloaded, and duplicates were removed. Initially, the title and abstract were reviewed to select only relevant studies from mainland China. Further article selections were performed by three researchers (HB, MS, and BH, independently) based on the full-text review of each article following the inclusion and exclusion criteria.

### Inclusion criteria

The included articles had at least ten *Candida* species isolated from clinical specimens and mentioned the total number of isolates. The articles had data on antifungal susceptibility tests (AST), written in English and published from December 2011 till December 2021 in mainland China.

### Exclusion criteria

All the articles were excluded, with isolates size less than ten, isolates not from human origin, did not perform the susceptibility tests or incomplete data, articles that did not use the standard authenticated protocols for AST, and review articles.

### Data extraction

The three researchers, HB, MS, and MNK, separately collect the data from each article into a pre-prepared Excel sheet (2016). The collected data were about publication year, location of the study, sampling duration, inpatient or outpatient, gender, age, infection types, species type, total number of isolates, identification method, AST method, breakpoint reference guidelines, tested drugs, susceptibility profile, mortality rate, the molecular mechanism of AFR, and molecular phylogeny of the *Candida* isolates. The duration of the samples was determined by the year in which the sampling concluded. All the patients aged less than 18 years were considered paediatric. Only the susceptible or wild-type isolates percentages were counted, and the intermediate or susceptible dose-dependent were not considered.

### Statistical analysis

The number of occurrences and percentages for each variable were counted in Excel sheet. The antifungal susceptibility pattern for each *Candida* species against every antifungal agent in the form of median susceptible or median wild type (MS/WT) with a 95 % confidence interval (CI) was determined to estimate a consistent ratio for combined data. The student’s *t*-test or the Wilcoxon signed-rank test was performed to measure the statistical significance of the antifungal susceptibility data against each drug. The *P*-values smaller than 0.05 were considered significant, calculated for two-tailed with a Gaussian approximation. For *C. albicans, C. tropicalis, C. glabrata, C. parapsilosis*, and *C. krusei*, due to isolation from different infection types, subgrouping M.S/WT, with 95% CI were also calculated. For each species, three subgroups were compiled i.e. isolates from all publications, invasive candidiasis and bloodstream infection (IC/BSI), and vulvovaginal and oral candidiasis (VVC/OC). Additionally, subgroup analysis was performed for different susceptibility testing methods i-e., BMD, ATB fungus 3, Sensititre YeastOne kit, and agar diffusion methods. The statistical analysis and graph designing were performed using the GraphPad Prism *v*8.0.2 software. Three researchers performed the calculation separately to negotiate any possible bias.

## Results

### Studies characteristics

Eighty-nine articles were included for systematic analysis containing 44,716 *Candida* isolates. High proportion of articles was published in 2019 and 2020 (13 in each), while for the duration of sampling, 40 studies were reported from 2010 to 2015 (supplementary file; figure S1). The maximum studies were reported from east China (*n* = 43), followed by north China (*n* = 33). Among the subregions, the highest number of articles were from Beijing (*n* = 20) and Shanghai (*n* = 18), while we did not find any study from two subregions of southwest China, i-e., Guizhou (province) and Tibet (autonomous region), and one province (Qinghai) of the northwest region (Supplementary file: figure S2). The number of articles stated the data about patient demography and AST are presented in Table 1. The median age with 95% CI from the ages mentioned articles (*n* = 47) was 57.45 years (53–62.5). The all-causes mortality rate was mentioned in 18 studies in which *C. albicans* associated median mortality was 27.3%, interquartile range (IQR) (13.60–38.30%). The median mortality rates (IQR) of *Candida* pathogens in included literature are stated in supplementary file (figure S3). Molecular typing of *Candida* isolates was performed only in 16 (17.98%) articles. The three different genotyping methods, i-e multilocus sequence typing (MLST), random amplified polymorphic DNA (RAPD) analysis, and microsatellite markers typing, were used in these studies. The detail about the molecular typing methods for the genotyping of *Candida* species is presented in Table 2.

**Table 1:** Characteristics of articles include in current systematic analysis (*n* = 89)

Characteristics	Frequency (%)	References
Species		
*C. albicans*	65 (73.03%)	(14, 26, 27, 37-42, 44, 47-54, 56-60, 67-108)
*C. tropicalis*	47 (52.81%)	(14, 37-43, 47-49, 51, 52, 54, 57-60, 67-76, 93, 94, 97-102, 104, 107-117)
*C. glabrata*	43 (48.31%)	(14, 17, 37-40, 42-44, 47-52, 56-59, 67-76, 96-102, 104-106, 108, 118-120)
*C. parapsilosis*	37 (41.57%)	(5, 14, 37-41, 43, 48, 49, 51-58, 60, 71-76, 93-95, 97-102, 108, 118, 121)
*C. krusei*	16 (17.98%)	(14, 38, 43, 44, 56, 59, 97, 99-101, 104-106, 108, 122, 123)
*C. guilliermondii*	6 (6.74%)	(14, 97, 98, 108, 124, 125)
*C. pelliculosa*	3 (3.37%)	(98, 102, 108)
*C. lusitaniae*	3 (3.37%)	(14, 97, 108)
*C. auris*	2 (2.25%)	(16, 24)
*C. haemulonii*	1 (1.12%)	(126)
*C. africana*	1 (1.12%)	(103)
Patient type		
Inpatients	35 (39.32%)	(16, 24, 26, 37-41, 47-54, 57-60, 68, 71, 72, 76, 89, 93, 102, 104, 105, 107, 111, 113, 115, 116, 118, 121)
Outpatients	3 (3.37%)	(70, 80, 96)
Both	12 (13.48%)	(14, 41, 55, 69, 94, 97, 98, 108, 110, 119, 120, 124)
NM	39 (43.82%)	NA
Age group		
Adult	42 (47.19%)	(5, 24, 26, 37, 39, 40, 42, 44, 49, 51-54, 56, 57, 59, 60, 67, 69-72, 75, 78-80, 84, 85, 90, 91, 93, 95, 96, 100, 103, 106, 107, 115, 118-120, 124)
Adult/ Pediatrics	18 (20.22%)	(14, 17, 38, 47, 48, 50, 55, 58, 76, 94, 97-99, 101, 102, 108, 110, 121)
Age group NM	29 (32.58%)	NA
Gender		
Female	19 (21.34%)	(26, 44, 69, 70, 75, 77-80, 85, 89-91, 95, 96, 100, 103, 106)
Male/Female	46 (51.68%)	(5, 14, 17, 24, 37-40, 42, 47-60, 67, 71, 72, 76, 87, 88, 93, 94, 97, 99, 102, 104, 107, 108, 110, 113, 115, 116, 118-121, 124)
NM	24 (26.97%)	NA
Infection types		
Invasive Candidiasis	27 (30.33%)	(5, 14, 37, 41, 43, 47, 49, 50, 59, 60, 68, 73, 74, 94, 97-99, 102, 105, 108, 114, 115, 117, 120, 123-125)
Bloodstream infection	15 (16.85%)	(17, 39, 40, 48, 51-55, 57, 58, 71, 72, 76, 93)
Vulvovaginal candidiasis	20 (22.47%)	(26, 44, 69, 70, 75, 77-80, 83-85, 89-91, 95, 96, 100, 103, 106)
Oral candidiasis	5 (5.61%)	(42, 56, 67, 104, 107)
Multiple infections	8 (8.99%)	(16, 86, 87, 109-111, 116, 121)
UTI	1 (1.12%)	(113)
Not Mentioned.	13 (14.61%)	NA
Phenotypic identification methods		
CHROMagar media	41 (46.06%)	(26, 37, 42, 44, 47, 50, 55, 56, 59, 67-70, 72, 75-83, 85-88, 91, 95, 96, 98, 99, 101-103, 105, 106, 109, 111, 112, 123)
MALDI-TOF MS	29 (32.58%)	(5, 16, 24, 38, 40, 43, 47, 48, 50, 57, 59, 67, 68, 80, 94, 97, 99, 102, 108, 110, 111, 113, 114, 116, 120, 121, 123, 124, 126)
API 20C AUX	21 (23.59%)	(26, 37, 41, 42, 55, 56, 70, 75, 77, 78, 83, 85, 87, 88, 95, 98-101, 105, 122)
VITEK 2 COMPACT	17 (19.10%)	(39, 41, 51-54, 58, 60, 67, 69, 72, 73, 76, 82, 96, 99, 106)
BD BACTEC™ FX	5 (5.61%)	(39, 40, 52, 53, 93)
VITEK MS system	2 (2.24%)	(14, 16)
Microscopy	2 (2.25%)	(85, 103)
DL-96A ID/AST	2 (2.25%)	(71, 118)
yeast identification kit	1 (1.12%)	(109)
ATB ID32C strips	1 (1.12%)	(27)
Brilliance *Candida* agar	1 (1.12%)	(41)
NM	4 (4.49%)	NA
Molecular identification methods		
ITS sequencings	32 (35.95%)	(5, 14, 16, 24, 38, 41-43, 55, 67, 72-74, 79, 84, 85, 90, 92, 94, 97, 99, 102, 104, 107, 108, 110, 115, 120, 123-126)
D1/D2 analysis	6 (6.74%)	(24, 37, 41, 73, 84, 110)
Molecular beacon assay	1 (1.12%)	(96)
Susceptibility testing method		
Broth microdilution	35 (39.32%)	(14, 17, 26, 56, 60, 67, 72-74, 77, 79-83, 85, 86, 89-92, 97, 99-101, 106, 109, 112-116, 120, 122, 123)
ATB FUNGUS 3 kit	24 (26.97%)	(27, 37, 39, 40, 47, 49-54, 57-59, 68-70, 78, 87, 88, 93, 96, 105, 119)
Sensititre Yeast-1 kit	16 (17.98%)	(5, 16, 24, 38, 42, 43, 48, 94, 102, 110, 111, 117, 120, 121, 124, 126)
Agar Diffusion method	12 (13.48%)	(41, 44, 75, 76, 95, 98, 103, 104, 107, 108, 110, 118)
ETEST method	2 (2.25%)	(37, 55)
kit of Autobio	1 (1.12%)	(71)
Neo-Sensitabs	1 (1.12%)	(84)
Breakpoint reference guidelines		
CLSI	82 (92.13%)	(5, 14, 16, 17, 24, 26, 27, 37-43, 47-52, 55-57, 59, 60, 67-70, 72-75, 77-83, 85-126)
EUCAST	3 (3.37%)	(40, 52, 93)
NM	7 (7.86%)	NA

*Footnote*: The sum of percentages is not equal to 100 because some studies stated more than one variable, and we counted each study separately with each variable. NM= Not mentioned, NA= Not applicable, UTI= urinary tract infection, ITS=internal transcribed spacer, CLSI= clinical laboratory and standard institutes, EUCAST= European committee of antimicrobial sensitivity testing

**Table 2:** The molecular typing of *Candida* species mentioned in the included studies

Species/ Method	Outcomes/Detail	References
*C. albicans*		
MLST	DSTs: 79, 435, 1867, 365, 1395, 254, 443, 365, and CC 69	(26, 27)
Microsatellite markers	CAI and CP6	(37, 72)
RAPD analysis	Primer: 5’-ACGGCCGACC-3’ and 5’-CCAGATGCAC-3’	(78, 87)
*C. tropicalis*		
MLST	DSTs; 225, 639, 329, 615, 506, 508, 519, 169, 345, 399, 300, 546, 376, 321, 322, 323, 324, 325, 114, 169, 326, 327, 328, 331, 332, 333, 334, 335, 336, 337, 338, 339, 340, 341, 164, 341, 342, 343, 344, 346, 347, 348, 349, 99, 350, 351, 352, 353, 354, 355, 356, 181, 615, 852, 984, 996, 991,482. 901, 330, 980, 337, 522, 990, 982, 998, 723, 983, 997, 977, 730, 489, 993, 403, 184, 139, 833, 889, 999, 995, 992, 986, 434, 981, 994, 394, 994, 437, 978, 525, 985, 923, 979, 986, 987, 988, 1000, 1001, 1002,	(72, 111, 113, 115, 116)
Microsatellite markers	ctm1, ctm3, ctm24	(117)
*C. glabrata*		
MLST	STs: ST7, ST10, ST22, ST45, ST55, ST3,	(17, 72, 120)
Microsatellite markers	GLM5, RPM2, GLM4, ERG3, MTI, GLM6,	(17, 120)
*C. parapsilosis*		
Microsatellite markers	CAI, CP6, B5, CP1, CP4,	(72, 121)
*C. krusei*		
Microsatellite markers	33 markers were selected; Cakr001–Cakr033	(123)
*C. guilliermondii*		
Microsatellite markers	sc15, sc32, sc72	(125)

Footnote: DSTs; Diploid sequence types, ST; Sequence types, MLST; multilocus sequence type. RAPD; Randomly amplified polymorphic DNA.

### Prevalence of Candida species

**Figure 2.**
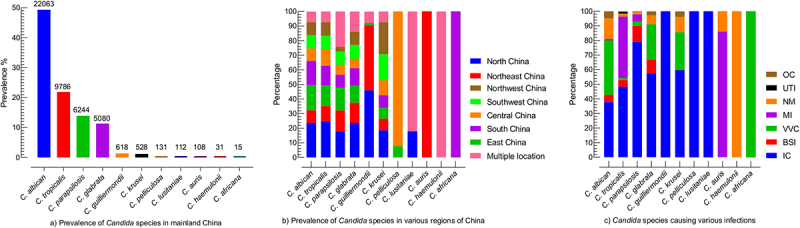
Prevalence of *Candida* species, (a) the total occurrence of Candida species, the numerical on the top of the bar is the number of specific *Candida* species, (b) the occurrence of *Candida* species in different regions of China, each region is represented by specific colour as shown in the box, Multiple locations mean the that studies mentioned more than one region of China, (c) Prevalence of *Candida* species in association with various infection type, OC; oral candidiasis, UTI; urinary tract infection, NM; not mentioned the infection type, MI; multiple infections, involved in more than one infections, VVC; vulvovaginal candidiasis, BSI; bloodstream infection, IC; invasive candidiasis.

*C.albicans* accounted for 49.36% of all *Candida* isolates reported in 65 articles. Among the non-*C. albicans* species, *C. tropicalis* (21.89%) was the most prevalent specie, followed by *C. parapsilosis* (13.92%) and *C. glabrata* (11.37%). *C. africana* (0.03%) was the most rarely reported *Candida* species. The percentages and number of all *Candida* species reported in the current study are presented in Figure 2(a). Broadly, mainland China is divided into seven regions, among which differences in the distribution of *Candida* species in various regions were observed. The highest number of isolates were reported from north China (23.07%), followed by east (15.20%), south (13.95%), northeast (11.12%), southwest (9.11%), central (8.69%), and northwest region (8.20%), while 10.64% of the isolates were from multiple locations. *C. albicans* and *C. tropicalis* were highly reported in the north (23.61% and 24.67%), followed by east (17.22%, and 14.08%) and south China (16.67%, and 13.57%). *C. parapsilosis* proportion was highest in the north (17.63%), followed by the east (15.63%) and northeast region (14.52%). *C. glabrata* was highest in the north (23.48%), northeast (13.99%), and south China (12.13%). *C. krusei* was high in the southwest (18.56%) while *C. auris* were only reported in northeast China. The complete depiction of *Candida* species distribution in various regions of China is presented in Figure 2(b).

Regarding infections type, the high proportion of *Candida* species was associated with invasive candidiasis (IC) (49.36%), followed by vulvovaginal candidiasis (VVC) (22.17%), bloodstream infections (BSI) (6.07%), and oral candidiasis (OC) (3.04%), while 10.31% of isolates were reported with multiple infections and for 8.09% strains the infections types were not mentioned. *C. albicans* were almost equally reported for IC and VVC (37.805% and 37.23% out of 22,063 isolates). *C. tropicalis* and *C. parapsilosis* were highly associated with IC (48.30%/9786 and 78.93%/6244). Similarly, *C. glabrata* and *C. krusei* were highly reported with IC (57.44% and 59.65%) followed by VVC (24.35% and 25.94%) out of 5080 and 528 isolates, respectively. Among 108 *C. auris* isolates, 86.11% of isolates were involved in multiple infection types, while for 13.89% of isolates, the infection types were not mentioned. The percentages of *Candida* species associated with various infection types are shown in Figure 2(c).

### Antifungal susceptibility pattern

The median susceptibility or wild-type (M.S/WT) with a 95% confidence interval (CI) was calculated for *C. albicans, C. tropicalis, C. glabrata, C. parapsilosis, C. krusei, C. guilliermondii, C. pelliculosa, C. lusitaniae*, and *C. auris*. The *C. haemulonii* and *C. africana* were reported in one study; therefore, their MS was not determined. The susceptibility rates of all antifungal agents were statistically significant (P = < 0.05) except few; terbinafine against *C. albicans* was not significant (*P* = 0.4164), voriconazole against *C. auris* (*P* = 0.4114), clotrimazole in case of *C. glabrata* (*P* = 0.1158), and clotrimazole, ketoconazole, and miconazole against *C. tropicalis* were not significant (*P* = 0.3308, 0.0574, and 0.1065, respectively). The *P*- values of antifungal agents against all tested *Candida* isolates are mentioned in supplementary file (table S1). For the subgroup analysis based on different susceptibility testing methods, it was observed that the susceptibility rates obtained by agar diffusion methods are comparatively low than BMD, ATB fungus 3, and Sensititre YeastOne kit method almost for all antifungal agents against all tested *Candida* species (supplementary file; table S2)

**Figure 3.**
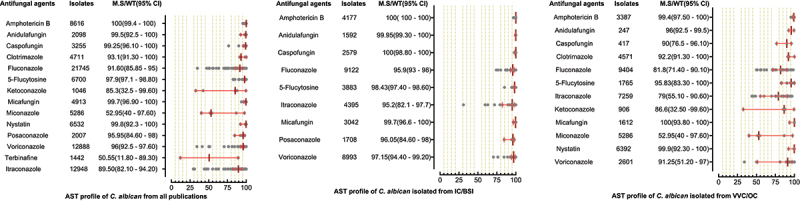
Antifungal susceptibility patterns of *Candida albicans* in the form of median susceptibility/wild type with 95% confidence interval.

**Figure 4.**
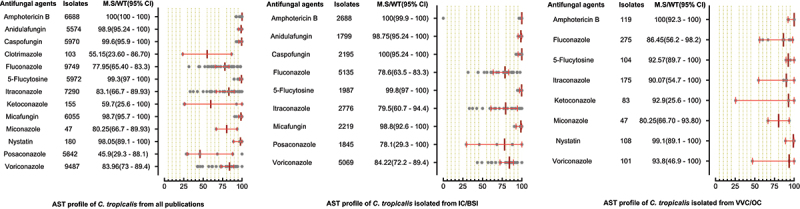
Antifungal susceptibility patterns of *Candida tropicalis* in the form of median susceptibility/wild type with 95% confidence interval.

AST of 22,063 *C. albicans* were performed against 14 antifungal drugs, in which high proportions of isolates were tested against fluconazole, itraconazole, voriconazole, and amphotericin B. The highest susceptibility was observed for polyenes, followed by echinocandins and 5-flucytosine. The azole class was comparatively less susceptible as the MS (95%CI) for miconazole, ketoconazole, and fluconazole were 52.95% (40–97.60), 85.3% (32.5–99.60), and 91.6% (85.85–95), respectively. In the IC/BSI group, *C. albicans* were tested against nine antifungal drugs, with susceptibility rates greater than 95% for all tested drugs. The micafungin and amphotericin B were the most susceptible drugs against *C. albicans* in VVC/OC group, having MS (95%CI) of 100% (93.80–100) and 99.4% (97.50–100), while the lowest susceptibilities were reported against miconazole (52.95% (40–97.60)) and itraconazole (79% (55.10–90.60)). AST pattern of *C. albicans* against all tested drugs and subgroups analysis is presented in Figure 3. AST of *C. tropicalis* was examined against 13 antifungal drugs, in which 100% susceptibility was reported against amphotericin B, while posaconazole (MS; 45.95, 95%CI; (29.3–88.1)) was less susceptible among all tested drugs. The susceptibility against echinocandins was in the range of 98–99%. Among the azole class, the highest MS (95%CI) was observed for voriconazole (83.96%, (73–89.4)) while fluconazole (77.95%, (65.4–83.3%)) was among the less susceptible drugs. For 5-flucytosine, variations in susceptibilities were reported between IC/BSI and VVC/OC groups, in which the MS (95% CI) were 99.8% [68–71] and 92.57% (89.7–100), respectively. The AST pattern of *C. tropicalis* against all antifungal drugs is depicted in Figure 4.

**Figure 5.**
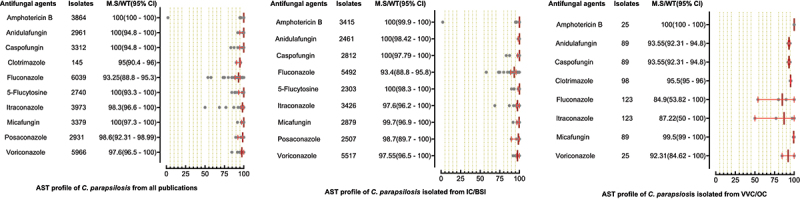
Antifungal susceptibility patterns of *Candida parapsilosis* in the form of median susceptibility/wild type with 95% confidence interval.

**Figure 6.**
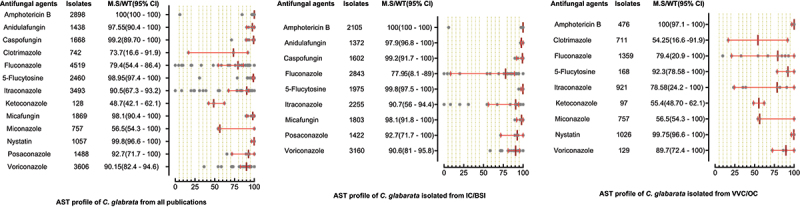
Antifungal susceptibility patterns of *Candida glabrata* in the form of median susceptibility/wild type with 95% confidence interval.

**Figure 7.**
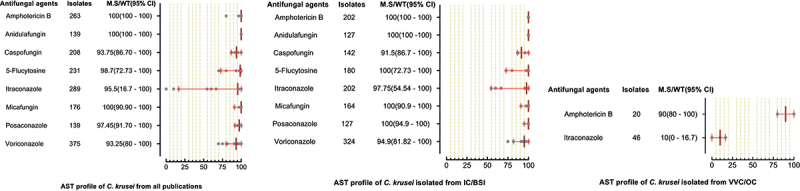
Antifungal susceptibility patterns of *Candida krusei* in the form of median susceptibility/wild type with 95% confidence interval.

**Figure 8.**
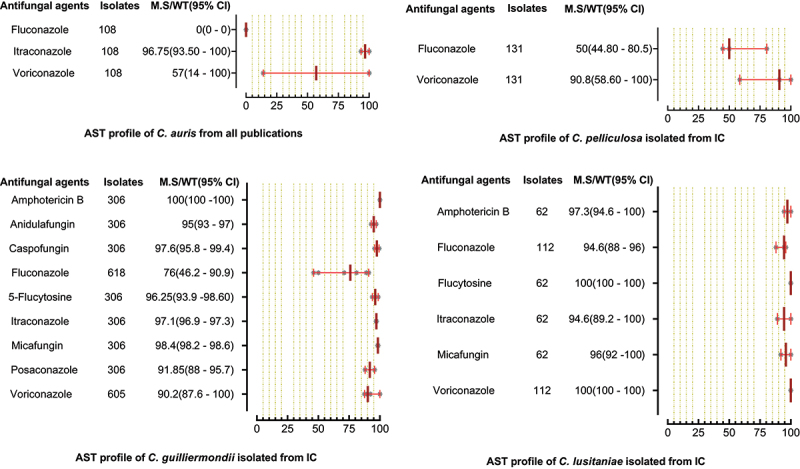
Antifungal susceptibility patterns of *C. auris, C. pelliculosa, C. guilliermondii* and *C. lusitaniae* in the form of median susceptibility/wild type with 95% confidence interval.

A total of 6244 *C. parapsilosis* isolates were examined for AST and showed the highest susceptibility rates to polyenes and echinocandins compared to the azole class. The susceptibilities of *C. parapsilosis* against all drugs were comparatively high than other *Candida* species. None of the isolates was resistant to amphotericin B, while the MS (95% CI) against fluconazole was 93.25% (88.8–95.3). The susceptibilities of the VVC/OC group containing *C. parapsilosis* against echinocandins and azole drugs were comparatively low than isolates of the IC/BSI group (Figure 5). For *C. glabrata* the lowest MS (95%CI) was reported against ketoconazole (48.7% (42.1–62.1)), followed by clotrimazole (73.7% (16.6–91.9)) and fluconazole (79.4% (54.4–86.4)), while was 100% susceptible against amphotericin B. Overall, the *C. glabrata* of the IC/BSI group were more susceptible to antifungal drugs than VVC/OC group containing isolates. The complete antifungal susceptibility pattern of *C. glabrata* is presented in Figure 6. *C. krusei* were 100% susceptible against amphotericin B and to two drugs of echinocandin class i-e., anidulafungin and micafungin, while the lowest MS (95%CI) were observed against caspofungin (93,75% (86.70–100). Similarly, in IC/BSI group, the lowest MS (95%CI) was reported against caspofungin (91.5% (86.7–100)), while other echinocandins, 5-flucytosine, and polyene were 100% susceptible (Figure 7). Among the rare *Candida* species, the lowest MS (95% CI) was found against fluconazole, which was 94.6% [59] - [67], 76% (46.2–90.9), 50% (44.80–80.5), in the case of *C. lusitaniae*, C. *guilliermondii*, and *C. pelliculosa* respectively. None of the *C. auris* isolates was susceptible to fluconazole, while the highest MS (95%CI) was observed against itraconazole (96.75% (93.50–100)). The complete susceptibility pattern of *C. guilliermondii, C. pelliculosa, C. lusitaniae*, and *C. auris* is presented in Figure 8.

### Molecular mechanism of antifungal resistance

Among the selected articles, 25 studies reported mutations in ergosterol biosynthesis genes lead to azole resistance. Only two studies reported echinocandins resistance due to *FKS1* mutation in *C. auris* and *FKS2* mutation in *C. glabrata* [90,100]. The gene mutations were detected mainly by PCR amplification and sequence analysis, except for one study that performed the whole-genome sequence of *C. auris* [100]. Resistance to azole due to the overexpression of regulatory genes was also detected in some studies by qRT PCR. The overall detail about the mutations in various genes, leading to amino acid substitutions and overexpression of genes causing the antifungal resistance in different *Candida* species from the included literature are mentioned in Table 3.

**Table 3:** Molecular mechanisms of antifungal drug resistance stated in the included studies

Mechanism	Detail	Outcome	Reference
*C. albicans*			
*ERG11* mutation	**Amino acid substitution**: A114S*, Y132H*, F449Y*, Q474K*, T123I*, Y257H*, G448E*, G464S*, F72S*, G450E*, Y132F*, K143Q*, K143R*, Y257H*, D116E, P56S, V88I, V437I, E266D, V488I, D116E/D, R523G, N440S, A114V, T285A, S457P, I333T, S24G, K90R, N168S, V234G, N237D, E260G, T285A, A317T, K344E, E517V, V332L, K128T, G448R, V488I, G448R/G, G465S, D225H, S199N, F104V, L935S, E260V, N435V, G472R and D502E, D117E, E165K, E174A, V234F, G346A, A434V, and L480F	Azole resistance	(26, 27, 70, 74, 80, 82, 83, 86, 88, 125)
*Mrr2* mutation	**Amino acid substitution**: L144V*, T145A*, V451A*, S466L*, A468G*, S480P*, H31Y, T83A, H149Y, S165N, T386I and D404N	Azole resistance	(85)
*ERG4* mutation	**Silent mutation**: CA133 and C435T	Azole resistance	(86)
*ERG3* mutation	**Amino acid substitution**: A18P/A, R365G/R, W219C and R352H **Silent mutations**: T342G, T435C, C441T, T1047C	Azole resistance	(26, 89)
*Efg1* mutation	**Amino acid substitution**: V86I **Silent mutation**: A150T, A165C, G210A, G267A, G279A, A285T, A744C, A786G, T954A, C1071T, A1055C, A1317G	Azole resistance	(89)
*Cap1* mutation	**Amino acid substitution**: A390T, S381N, P311S, G481E	Azole resistance	(90)
*Mrr1* mutation	**Amino acid substitution**: R557K*, K844E*, F1032L*, S1037L*, N937K, E1020Q, T917M and T923I	Azole resistance	(90)
Overexpression	*ADH1, CDR1, CDR2, MDR1, FLU1, ERG^11^, TAC1, Cap1, MDR1, Mrr1*	Azole resistance	(26, 27, 77, 81, 85, 90, 106)
*C. glabrata*			
*ERG 11* mutation	**Mutations**: T1328C, T1394C, G1487A, A1583G Amino acid substitution= no	Fluconazole resistance	(119)
*MSH2* mutation	**Polymorphism**: V239L, E456D, R636 M	Azole resistance	(17)
*PDR1* mutation	**Amino acid substitution**: G348C*, N764D*, E259G, E555D, N764D, D876N, S98L, R250K, G389V, R293G, S942F, L139I, E818G, Y336H, I378T, L669F, A848V, P76S, P143T, D243N, I91V, L732S, P927S	Azole resistance	(17, 119)
*FKS2* mutation	S663F and S663P mutation in FKS2 HS1	Echinocandins resistance	(17)
Overexpression	CDR1, CDR2,	Azole resistance	(119)
*C. tropicalis*			
*ERG 11* mutation	**Amino acid substitution**: Y132F*, S154C*, A114S*, Y132H*, V125A, Y257H, and G464S	Azole resistance	(68, 94, 111, 112, 114)
Overexpression	Erg^11^, CDR1, MDR1	Azole resistance	(114)
*C. krusei*			
*ERG 11* mutation	**Codon substitution**: T939C, T642C, A756T	Azole resistance	(122)
Overexpression	Erg11	Azole resistance	(122)
*C. auris*			
*ERG 11* mutation	VF125AL and I74L mutation	Azole resistance	(16)
*FKS1* mutation	S639F mutation	Echinocandins resistance	(16)
*C. guilliermondii*			
*ERG 11* mutation	**Non-synonymous mutations**: W37C, P518R, P430Q, D492N, Y41F, L328T, S346T, V410M, S420T, F39L, N485K, Y132F, S346T, G16S, M332I, R247K, G459S, G459K, K143R, Q469K	Azole resistance	(125)
*C. haemulonii*			
*ERG 11* mutation	**Amino acid substitution**: Y132H*	Azole resistance	(94)

*= Validated: mean all the amino acids found only in resistant strains and not in the susceptible isolates, and the authors discussed their azole resistance validity.

## Discussion

The current study systematically analysed the distribution and AST pattern of *Candida* isolates from mainland China to provide reference points for upcoming studies. Among the selected articles, *C. albicans* was found in a high proportion (49.36% of all the isolates). *C. albicans* is a worldwide highly prevalent opportunistic pathogen [103]. Among the non-*C. albicans* species, *C. tropicalis* (21.89%) was the most prevalent, followed by *C. parapsilosis* (13.92%) and *C. glabrata* (11.37%). This drift contrasts with many European countries and North America, where the *C. glabrata* is more prevalent among the non-*C. albicans* isolates [104]. However, a similar trend of *Candida* species prevalence resembling our study was also reported in India, Nigeria, and Cameroon [105]. Among the infection types, a high proportion of *C. albicans* was associated with VVC. This is mainly due to the colonization of *C. albicans* in the human vagina, and getting the opportunity causes vaginal infection [106]. The invasive candidiasis and bloodstream infections are also reported in association with *C. albicans* and non-*C. albicans* species, The three main factors i.e. misuse of broad-spectrum antibiotics, cytotoxic chemotherapy-induced mucositis, and iatrogenic immunosuppression, are the causes of the high proportion of invasive candidiasis [107]. The rare *Candida* species were not explicitly observed with candidemia. However, the *C. guilliermondii*, *C. pelliculosa*, *C. lusitaniae*, and *C. auris* were reported to cause invasive candidiasis. This is mainly due to their MDR properties which lead to treatment failure and a longer duration of invasive candidiasis [108].

The overall *C. auris* clades reported in China belong to South Asian and South African clades [100,101]. The discovery of *C. auris* in multiple clades is mainly due to the increasing business exchange and global travelling in recent years, as reported previously in the United States [109]. In the included articles, only two studies performed the MLST of *C. albicans*, revealed that most of the strains belong to novel DSTs, which indicates that genetic diversity is largely unknown. However, most of the DSTs known strains belong to clades 1 and 18, suggesting their nosocomial dissemination [16,17]. Interestingly, the worldwide distributed clade of *C. albicans* is clade 2, which is not identified in China [17,110]. It might be due to the highly distinct global distribution of *C. albicans* clades. Furthermore, it is also reported that different body sites are associated with different clades, like *Candida* of superficial infections mostly belongs to clade 1 and BSI from clade 4 [17]. Further studies regarding molecular epidemiology targeting different body sites and populations are required.

The summary of the antifungal susceptibility profile showed that testing of azole, echinocandins, and polyenes is standard in China. In most *Candida* species, low susceptibility was observed for the azole group. Like in *C. albicans* the lowest susceptibility rates were observed for miconazole, ketoconazole, and fluconazole; 52.95%, 85.3%, and 91.60%, respectively. Similar trends of lowest susceptibility trend of azoles were also observed for non-*C. albicans* species. For *C. tropicalis*, the fluconazole and itraconazole susceptibility rates were 77.95% and 83.1%, respectively. Likewise, the fluconazole susceptibility rates for *C. glabrata* and *C. parapsilosis* were 79.4% and 93.25%, respectively. The *C. glabrata* and *C. tropicalis* are considered to have low susceptibility against azole worldwide. In the United States, Australia, and several European countries, 85% to 94% of *C. glabrata* isolates were reported susceptible to fluconazole [1,111,112]. In the United States, the *C. tropicalis* isolates were >97% susceptible to fluconazole [113]. For Latin America, Asia -Pacific regions, and Chile, nearly 90% of *Candida* isolates were azole susceptible [1,14,111]. The azole susceptibility of the current study is lower compared to other regions of the world and from tested polyenes and echinocandins drugs in the present study. This might be due to the high use of azole drugs in China, leading to molecular alteration of ergosterol biosynthetic pathways [114,115]. The reasons for their often prescriptions are that they are economical, available for oral administration, and reveal less toxicity [116]. The *C. auris* was reported in only two studies, which showed 100% resistance to fluconazole and 57% susceptibility to voriconazole. The highest susceptibility to amphotericin B was observed for all *Candida* species. It is mainly due to its less prescription as it is costly and causes severe renal toxicity [117]. The rapid emergence of echinocandins resistance has been observed worldwide, owing to their high use. In the United States, > 10% of *C. glabrata* isolates showed resistance to echinocandins [3]. In the current study, the echinocandins resistance is relatively low (0.8–2.5%) against *C. glabrata*. Their accurate prescriptions and usage must be maintained as the chances of acquiring resistance to echinocandins due to mutations in hotspot regions of *FKS* are very high [118]. The variation in susceptibility pattern of certain *Candida* species against azole drugs depends on various factors. The susceptibility reported in the early years was high compared to detected later in 2020 and 2021, which indicates that the susceptibility decreases due to misuse of azole drugs. For example, a study reported 85% fluconazole susceptibility in 2013, while in 2020, 48% susceptibility was reported against *C. glabrata* [18–21]. Likewise, a study for *C. parapsilosis* in 2012 reported 98.6% susceptibility, while 76.93% susceptibility was reported in 2020 against itraconazole [22,23]. Likewise, a study in 2014 reported 100% itraconazole susceptibility rate against *C. krusei*, while in 2016, a study reported 16.7% susceptibility rate [24,80]. Also, we reported that the median susceptibility values obtained by agar diffusion methods are lower than other methods in most cases (Supplementary table S2). Our finding contradicts earlier studies in 2002 and 2007, as they reported the same results for agar diffusion and other methods [45,119]. Further comparative studies need to evaluate the results of antimicrobial susceptibility methods based on the molecular known susceptible and resistant magnitudes. Additionally, many other factors like geography, study population, usage of azole drugs, and isolates obtained from various body sites cause variation in the anti-azole susceptibility pattern among various studies [46, 120].

Many samples were isolated from the long-term ICU, and hospitalized patients indicated that nosocomial disease is an important predisposing factor for candidiasis [20,25–31,101]. Besides this, the co-morbidities like hypoproteinemia, cardiovascular disease, diabetes, respiratory dysfunction, renal failure, cancer, HIV, and thrombocytopenia are reported to be significant for candidiasis development [21,27,29,30,32,33,94]. Apart from this, catheterization, mechanical ventilation, elderly patients, empirical antifungal therapy, and surgeries are secondary risk factors [21,26,27,29–37,94,101]. The risk factors reported in the present study are comparable to the findings of other studies [105,61,121,122]. The health care workers need special attention to hand washing or decontamination and provide an aseptic environment to minimize the horizontal transmission of candidiasis in the hospital.

The overall proportion of molecular studies regarding the genotyping and resistant mechanisms was unsatisfactory. The data obtained from molecular analysis give in-depth knowledge about the epidemiological origin and source of infections. Based on this, the health official eradicates the pathogens from their origins and inhibits their emergence and transmissions. Moreover, the molecular AFR study is essential to understand the intrinsic and acquired resistance mechanism, which may help to prevent the resistance and to design alternative and novel therapeutic agents [63,123]. To provide new insights, further molecular studies with a precise mode of action through genotyping and resistant mechanisms are required to understand pathogenicity and resistant magnitude.

Many gaps in the surveillance studies were noted, i.e. we did not find any study according to our inclusion criteria from Qinghai and Tibet Autonomous Region. The mortality rate was not mentioned in 79.77% of the studies. Nine studies did not mention the mortality rate concerning specific *Candida* pathogen. Similarly, 43.82% of the studies did not mention the patient types, as the inpatients are more vulnerable to candidiasis. Moreover, 47.19 %, 26.97%, 7.86%, and 4.49% of the studies did not mention the age, gender, breakpoint reference guidelines, and identification method, respectively. More importantly, such gaps in the studies make the data suspicious and difficult to analyse for practical applications. We recommend that the researchers fulfill these gaps in their future studies.

The current study focuses on the antifungal susceptibility pattern of *Candida* species from mainland China; however, its implication is worldwide. It is well known that resistant species are rarely limited to specific locations; any area with high drug resistant strains can act as a reservoir, from which the resistant species can be transmitted to other parts of the globe via humans, water, agricultural products, and animals [64,124]. Moreover, the current study is a point of reference for subsequent studies, as we identified the gaps in surveillance studies that need to be addressed in future studies. The information provided here may help for the development of treatment recommendations in the future that may need to be regionally tailored. Additionally, this study would dictate the overall AST profiles of *Candida* species, which will help the policymakers and health care officials to eradicate and exert a curative effect of antifungal agents dealing with candidiasis in mainland China.

### Limitations

Most of the studies included in this systematic report were from Beijing (*n* = 20) and Shanghai (*n* = 18) regions; there is a risk of selection bias. However, Beijing is political, and Shanghai is the economic hub of China, and people from all around the country are directly or indirectly connected with these two cities. There is also a chance of bias due to the not mentioned patient type in numerous studies; usually, the *Candida* species isolated from inpatients are less susceptible than outpatient [65,125]. In addition, it would be preferable to include studies that contain at least 30 isolates to ensure high-quality data accuracy [66,126]. However, due to the fewer existing studies, inclusion criteria regarding the isolates in each study had set at ten to cover high number of studies. Furthermore, data acquired through various methods from diverse patient groups were assembled in the present study. However, many studies used microdilution methods and followed CLSI guidelines; the degree of variation should be marginal.

## Conclusion

This study summarizes the antifungal susceptibility pattern of *Candida* species from mainland China and found that azole had the lowest, while amphotericin B and anidulafungin had the highest susceptibility rates among the tested antifungal drugs. The clinician can select their empirical antifungal therapy based on the outcomes of the current study. We noted substantial gaps in the surveillance studies, like no studies were found in Qinghai and Tibet regions among the included articles. Also, the number of articles for some *Candida* species was insufficient to calculate their susceptibility pattern. Furthermore, fewer studies performed genotyping and molecular analysis of antifungal resistance. Therefore, continuous molecular surveillance studies by researchers focusing on the mentioned gaps are of paramount importance in combating candidiasis. Along with that, precautionary measures from health staff following the guidelines of health care policymakers are necessary to halt the nosocomial dissemination and antifungal drug resistance.

## Supplementary Material

Supplemental Material

## Data Availability

Data supporting our study can be provided by demand from the corresponding author (email: zeng_yb@163.com) or the first author (email: bilal.microbiologist@yahoo.com).
