# Supplementary material for: Distribution and antifungal susceptibility pattern of Candida species from mainland China: A systematic analysis
Source: Virulence. 2022 Sep 18;13(1):1573–89. doi: 10.1080/21505594.2022.2123325 (PMC9487756; doi:10.1080/21505594.2022.2123325)
Supplement: Supplemental Material [file KVIR_A_2123325_SM0291.pdf]

Supplementry file:

Distribution and antifungal susceptibility pattern of *Candida* species from mainland China: A systematic analysis

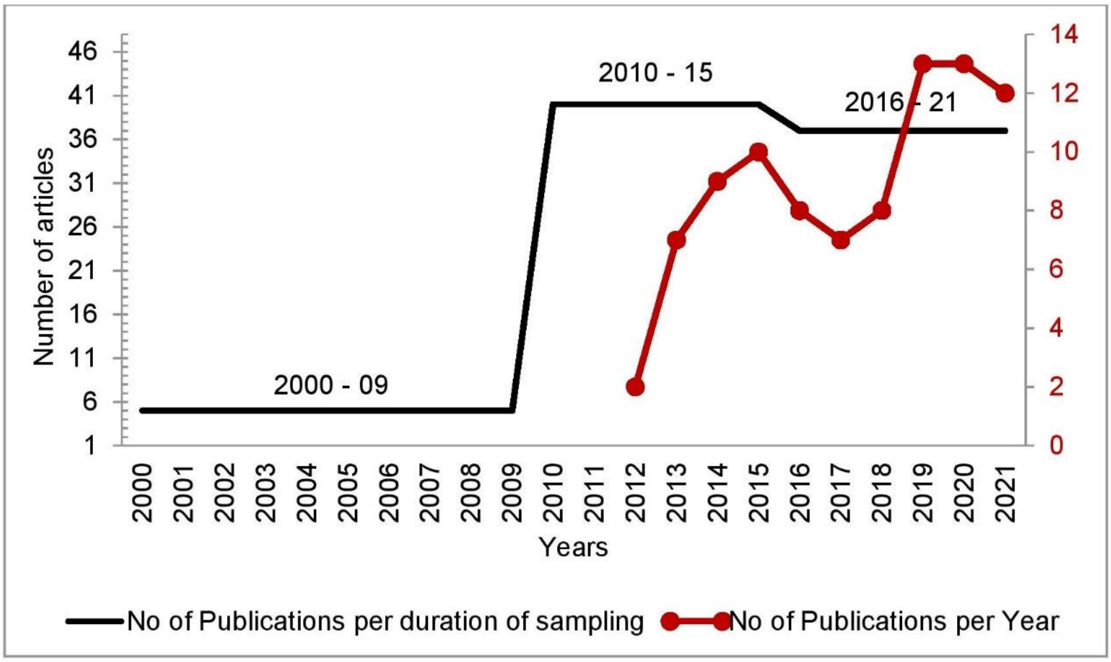

Figure S1 The number of articles based on sampling duration and publication per year

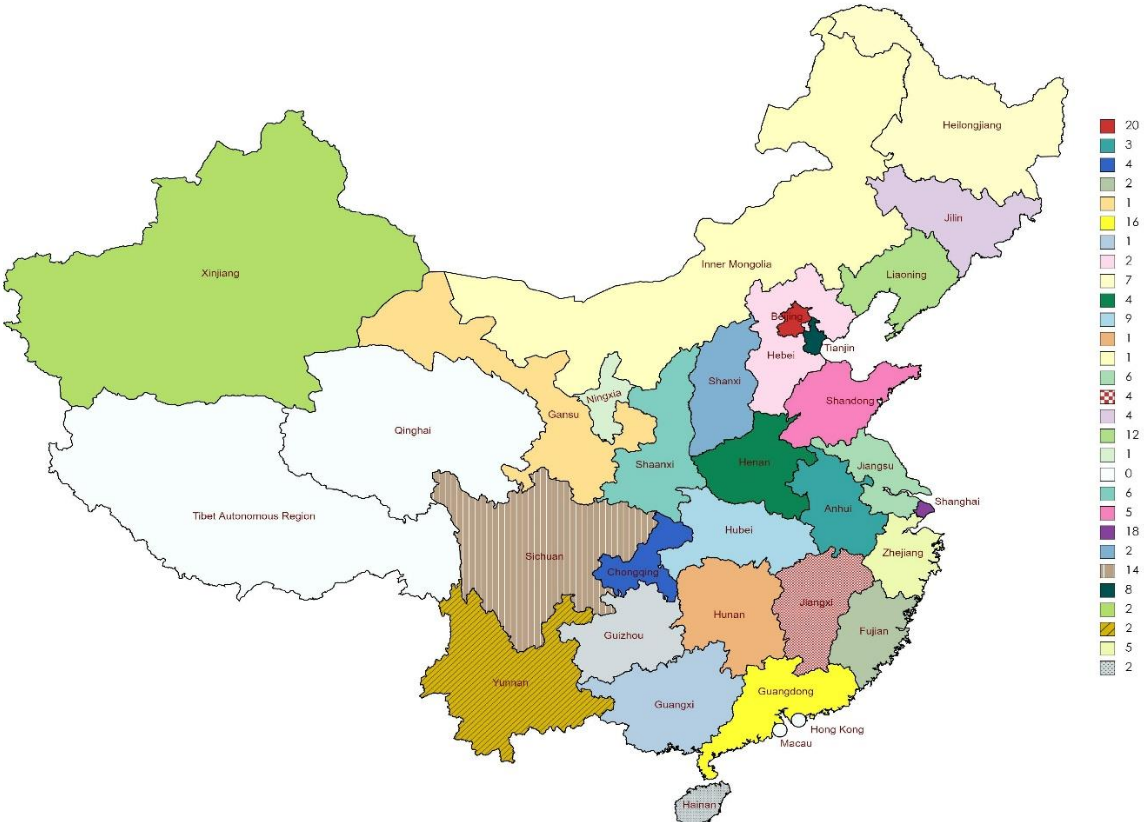

Figure S2 The number of articles from the different regions of mainland China included in present study. The names of region are written inside the map and number of studies from each region is shown by the different colors as shown in the boxes outside the map.

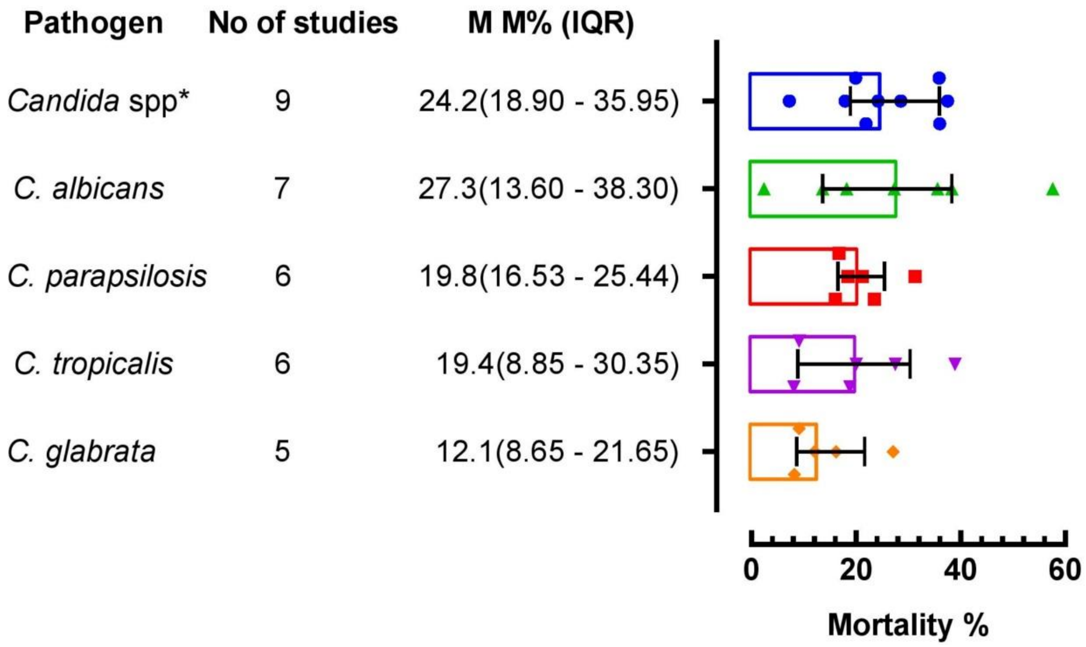

Figure S3 The median mortality rate with interquartile range of *Candida* pathogens, *= not specified the *Candida* pathogen type.

Table S1 *P*-values of the susceptibilities rate of all antifungal drugs against *Candida* species included in current study.

C. albicans

Drugs	P value	Significant
Amphotericin B	<0.0001	Yes
Anidulafungin	<0.0001	Yes
Caspofungin	<0.0001	Yes
Clotrimazole	<0.0001	Yes
Fluconazole	<0.0001	Yes
5-Flucytosine	<0.0001	Yes
Itraconazole	<0.0001	Yes
Ketoconazole	0.0066	Yes
Micafungin	<0.0001	Yes
Miconazole	0.0016	Yes
Nystatin	<0.0001	Yes
Posaconazole	<0.0001	Yes
Voriconazole	<0.0001	Yes
Terbinafine	0.4164	No

C. glabrata

Drugs	P value	Significant
Amphotericin B	<0.0001	Yes
Anidulafungin	<0.0001	Yes
Caspofungin	<0.0001	Yes
Clotrimazole	0.1158	No
Fluconazole	<0.0001	Yes
5-Flucytosine	<0.0001	Yes
Itraconazole	<0.0001	Yes
Ketoconazole	0.0131	Yes
Micafungin	<0.0001	Yes
Miconazole	0.042	Yes
Nystatin	<0.0001	Yes
Posaconazole	<0.0001	Yes
Voriconazole	<0.0001	Yes

C. tropicalis

Drugs	P value	Significant
Amphotericin B	<0.0001	Yes
Anidulafungin	<0.0001	Yes
Caspofungin	<0.0001	Yes
Clotrimazole	0.3308	No
Fluconazole	<0.0001	Yes
5-Flucytosine	<0.0001	Yes
Itraconazole	<0.0001	Yes
Ketoconazole	0.0574	No
Micafungin	<0.0001	Yes
Miconazole	0.1065	No
Nystatin	<0.0001	Yes
Posaconazole	0.0003	Yes
Voriconazole	<0.0001	Yes

C. parapsilosis

Drugs	P value	Significant
Amphotericin B	<0.0001	Yes
Anidulafungin	<0.0001	Yes
Caspofungin	<0.0001	Yes
Clotrimazole	0.0003	Yes
Fluconazole	<0.0001	Yes
5-Flucytosine	<0.0001	Yes
Itraconazole	<0.0001	Yes
Micafungin	<0.0001	Yes
Posaconazole	<0.0001	Yes
Voriconazole	<0.0001	Yes

C. krusei

Drugs	P value	Significant
Amphotericin B	<0.0001	Yes
Anidulafungin	<0.0001	Yes
Caspofungin	<0.0001	Yes
5-Flucytosine	<0.0001	Yes
Itraconazole	<0.0001	Yes
Micafungin	<0.0001	Yes
Posaconazole	<0.0001	Yes
Voriconazole	<0.0001	Yes

C. guilliermondii

Drugs	P value	Significant
Anidulafungin	0.0134	Yes
Caspofungin	0.0117	Yes
Fluconazole	0.0003	Yes
5-Flucytosine	0.0155	Yes
Itraconazole	0.0013	Yes
Micafungin	0.0013	Yes
Posaconazole	0.0267	Yes
Voriconazole	<0.0001	Yes

C. auris

Drugs	P value	Significant
Itraconazole	0.0214	Yes

C. pilliculosa

Drugs	P value	Significant
Fluconazole	0.0344	Yes

Voriconazole	0.4114	No		Voriconazole	0.022	Yes

C. lustinane

Drugs	P value	Significant
Amphotericin B	0.0177	Yes
Fluconazole	0.0007	Yes
Itraconazole	0.0363	Yes
Micafungin	0.0265	Yes

Table S2 Median susceptibility plus 95% confidence interval (MS (95%CI) of antifungal drugs against Candida species obtained by different susceptibility testing methods.

Drug<br>AST method	*C. albicans*<br>MS (95% CI)<br>Tested isolates	*C. glabrata*<br>MS (95% CI)<br>Tested isolates	*C. tropicalis*<br>MS (95% CI)<br>Tested isolates	*C. parapsilosis*<br>MS (95% CI)<br>Tested isolates	*C. krusei*<br>MS (95% CI)<br>Tested isolates
**Amphotericin B**					
ATB Fungus 3 kit	100 (99.3 - 100)<br>*n* = 1945	100 (100 - 100)<br>*n* = 575	100 (100 - 100)<br>*n* = 505	100 (96.50 - 100)<br>*n* = 545	100 (100 - 100)<br>*n* = 23
BMD	100 (98.9 - 100)<br>5929	100 (99.8 - 100)<br>*n* = 1426	100 (99.9 - 100)<br>*n* = 1324	100 (99.90 - 100)<br>*n* = 1472	100 (96.6 - 100)<br>*n* = 178
Agar Diffusion	97.2 (95.5-98.8)<br>*n* = 236	90.9 (84.6 - 97.1)<br>*n* = 81	92.9 (92.30 - 99.8)<br>*n* = 3769	-	-
Sensititre YeastOne	100 (97.5 - 100)<br>*n* = 446	100 (97.4 - 100)<br>*n* = 774	100 (98.2 - 100)<br>*n* = 4792	100 (95.95 - 100)<br>*n* = 1775	100 (100 - 100)<br>*n* = 52
**5-Flucytosine**					
ATB Fungus 3 kit	97.6 (95.5 - 98.5)<br>*n* = 1564	100 (94.6 - 100)<br>*n* = 433	98.5 (94.6 - 100)<br>*n* = 306	100 (98.80 - 100)<br>*n* = 317	86.37 (72.73 -100)<br>*n* = 23
BMD	99.2 (97.1-100)<br>4394	99.50 (98.9 - 100)<br>1130	100 (98.8 - 100)<br>*n* = 1276	100 (93.10 - 100)<br>*n* = 1460	97.40 (80 - 100)<br>*n* = 146
Agar Diffusion	86.65 (83.3 - 90)<br>*n* = 236	60.9 (30.7 - 91.2)<br>*n* = 81	89.93 (89.7 - 99)<br>*n* = 3769	-	-
Sensititre YeastOne	98.1 (82.5-98.6)<br>*n* = 446	98.10 (78.6 - 100)<br>*n* = 774	99 (98.2 - 100)<br>*n* = 4285	93.3 (84.62 - 9.2)<br>*n* = 891	100 (100 - 100)<br>*n* = 52
**Fluconazole**					
ATB Fungus 3 kit	93 (87.5 - 96.1)<br>*n* = 2563	79.85 (20 - 88.80)<br>*n* = 595	70.3 (60 - 88.8)<br>*n* = 576	91.45 (87.8 - 100)<br>562	-
BMD	89.6 (63.3- 95.9)<br>*n* = 9008	90.60 (0 - 100)<br>*n* = 1132	78.9 (63.5 - 89.3)<br>*n* = 2468	93.95 (80 - 100)<br>*n* = 1514	-
Agar Diffusion	83.9 (72.6- 99.5)<br>*n* = 9330	32.90 (4.3 - 81.3)<br>*n* = 1789	81.7 (31.6 - 94.3)<br>*n* = 5491	84.9 (57.7 - 98.6)<br>*n* = 2035	-
Sensititre YeastOne	95 (55 - 99.30)<br>*n* = 584	79.70 (0 - 92.86)<br>*n* = 774	77.35 (45.5 - 88.4)<br>*n* = 4840	94.5 (53.8 - 97.7)<br>*n* = 1856	-
**Itraconazole**					
ATB Fungus 3 kit	88.1 (82.8- 96.3)<br>*n* = 2326	72.6 (30.8 - 93.2)<br>*n* = 556	63.9 (42.6 - 90.4)<br>*n* = 517	97.2 (87.2 - 100)<br>*n* = 562	60.6 (54.54 - 66.6)<br>*n* = 23
BMD	88.3 (48.9 - 95.8)<br>*n* = 5123	96.6 (24.20 - 100)<br>*n* = 1159	81.90 (58.6 - 97)<br>*n* = 1846	99.4 (50 -100)<br>*n* = 1472	96.60 (10 - 100)<br>*n* = 178
Agar Diffusion	90.6 (46.5- 93.6)<br>*n* = 4792	35.2 (8.1 - 90.6)<br>*n* = 804	86.5 (65.1 - 90.2)<br>*n* = 3761	97.5 (96.2 - 100)<br>*n* = 124	8.35 (0 - 16.70)<br>*n* = 36
Sensititre YeastOne	94.2 (6 - 99.3)<br>*n* = 446	92.3 (78.6 - 93.2)<br>*n* = 774	89.1 (78.1 - 100)<br>*n* = 4792	100 (76.9 - 100)<br>*n* = 1775	100 (100 - 100)<br>*n* = 52
**Voriconazole**					
ATB Fungus 3 kit	95.3 (88.5 - 97.9<br>*n* = 2438	94.60 (89.7 - 100)<br>*n* = 506	73 (61.10 - 9.40)<br>*n* = 549	97.45 (95.7 - 100)<br>*n* = 631	78.41 (75 - 81.82)<br>*n* = 23
BMD	96.3 (51.2- 98.3)<br>*n* = 5181	86.2 (58.4 - 95.3)<br>*n* = 1159	83.96 (49.2 - 92.9)<br>2407	100 (92 - 100)<br>*n* = 1514	93.40 (80 - 100)<br>*n* = 178
Agar Diffusion	99.3 (99.2 - 100)<br>*n* = 4515	85.2 (73.3 - 92.3)<br>*n* = 991	91.30 (79.7 -94.30)<br>*n* = 5393	100 (97.50 - 100)<br>1933	-
Sensititre YeastOne	95.9 (9 - 96.9)<br>*n* = 584	71.8 (36.50 - 100)<br>*n* = 774	79.30 (42.50 - 100)<br>*n* = 4818	95.90 (99.2 - 100)<br>*n* = 1856	84.85 (69.70 - 100)<br>*n* = 52
